# A Federated Multi-Agent Communication Framework for Autonomous Cyber Defense in Edge–Cloud IoT Environments

**DOI:** 10.3390/s26185775

**Published:** 2026-09-11

**Authors:** Hakan Aydin

**Affiliations:** Department of Computer Engineering, Faculty of Engineering and Architecture, Istanbul Topkapi University, Istanbul 34310, Turkey; hakanaydin@topkapi.edu.tr

**Keywords:** intelligent agent communication, multi-agent systems, federated learning, edge–cloud IoT, DDoS defense

## Abstract

With increasing system complexity and attack sophistication, autonomous cyber defense has become essential in edge–cloud Internet of Things (IoT) ecosystems, requiring intelligent, secure, and scalable agent communication frameworks. This paper proposes FMAD (Federated Multi-Agent Defense), a conceptual federated multi-agent framework that enables intelligent agent communication for autonomous cyber defense through collaborative sensing and federated learning, with a design emphasis on security and privacy preservation. The proposed framework defines a set of specialized intelligent agents responsible for distributed traffic sensing, threat intelligence analysis, federated model optimization, secure agent-to-agent communication, and adaptive mitigation. As an initial proof of concept, the Threat Intelligence Agent (TIA) was implemented using an xLSTM-based temporal intrusion detection model developed in this study and trained on the CIC-IoT-2023 dataset, achieving a detection accuracy of 99.89% while efficiently identifying temporal traffic anomalies at edge nodes. The FMAD framework integrates multiple collaborative agents for secure communication, federated learning, and coordinated cyber defense in edge–cloud IoT environments. The experimental evaluation focuses on the xLSTM-based Threat Intelligence Agent (TIA) for DDoS detection, while the remaining components are assessed at the design level. However, empirical validation in the current study is limited solely to the TIA. To support trustworthy collaboration among distributed agents, the framework further proposes a secure communication architecture based on Elliptic Curve Cryptography (ECC) key agreement, ChaCha20-Poly1305 authenticated encryption, and a permissioned blockchain ledger, which are designed to provide secure interaction management and traceability within the stated security assumptions. This federated multi-agent approach provides a promising foundation for intelligent agent communication and scalable autonomous cyber defense in real-world edge–cloud IoT environments.

## 1. Introduction

The rapid proliferation of the Internet of Things (IoT) has transformed traditional computing infrastructures into highly distributed edge–cloud ecosystems, where heterogeneous sensing devices, edge nodes, and cloud platforms collaboratively perform data acquisition, intelligent processing, and real-time decision-making [1,2,3]. By bringing computational resources closer to data sources, edge computing reduces communication latency, alleviates network congestion, improves bandwidth utilization, and enables real-time analytics for latency-sensitive applications [1,4]. Edge–cloud IoT systems have become a key enabling technology for numerous application domains, including smart cities, industrial automation, intelligent transportation, healthcare, and critical infrastructure monitoring. However, the increasing scale, heterogeneity, and dynamic connectivity of edge–cloud IoT ecosystems have significantly expanded their cyber attack surface, making security and cyber resilience essential for reliable and trustworthy system operation [2,5,6].

Among the diverse cyber threats targeting edge–cloud IoT environments, Distributed Denial-of-Service (DDoS) attacks remain one of the most disruptive because compromised IoT devices can be organized into large-scale botnets capable of exhausting communication bandwidth, computational resources, and service availability [7]. More recently, the emergence of artificial intelligence (AI)-assisted attack strategies has further increased the sophistication of cyber threats by enabling adaptive attack behaviors, automated reconnaissance, and dynamic evasion techniques, thereby reducing the effectiveness of conventional signature-based and centralized defense mechanisms. Consequently, large-scale edge–cloud IoT infrastructures require intelligent, distributed, adaptive, and autonomous cyber defense mechanisms beyond the capabilities of traditional security approaches. Although conventional intrusion detection approaches can achieve satisfactory detection performance under controlled conditions, they become increasingly inefficient in large-scale IoT deployments because of excessive communication overhead, increased detection latency, limited scalability, and privacy concerns [8,9,10]. Furthermore, centralized decision-making introduces potential single points of failure, reducing system resilience against coordinated and distributed cyber attacks [10]. These limitations have stimulated growing interest in distributed cyber defense paradigms that combine decentralized intelligence, privacy-preserving learning, and collaborative decision-making to improve both scalability and operational resilience.

**Figure 1:**
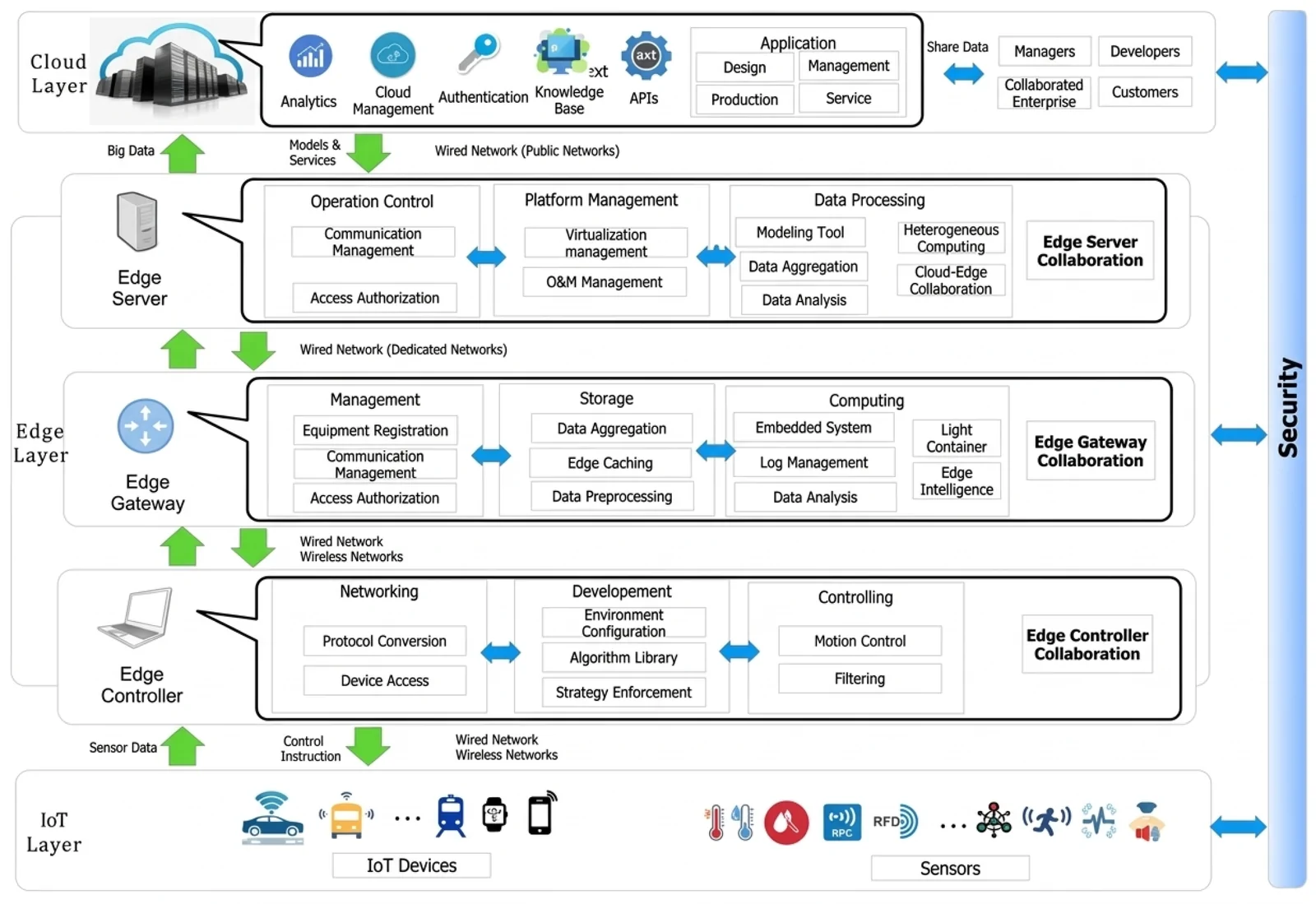
Edge–cloud IoT architecture and security challenges (reprinted from [11]).

Figure 1 illustrates a representative edge–cloud IoT ecosystem, highlighting the interactions among sensing devices, edge intelligence, cloud services, communication layers, and the major cybersecurity challenges that motivate the need for distributed autonomous cyber defense.

To overcome the limitations of centralized cyber defense, Multi-Agent Systems (MASs) have emerged as a promising paradigm for enabling distributed intelligence in dynamic and heterogeneous environments. As emphasized by Kott [12], autonomous cyber-defense agents should be capable of sensing their environment, collaborating with peer agents, learning from observations, and autonomously executing defensive actions in response to evolving cyber threats. Within a MAS, autonomous agents cooperate through intelligent agent communication by exchanging contextual knowledge, performing distributed reasoning, coordinating decisions, and executing collaborative actions without relying on a centralized controller [13]. By supporting distributed sensing, collaborative decision-making, adaptive response, and fault-tolerant operation, MASs provide a scalable and resilient foundation for next-generation autonomous cyber defense [14,15]. While distributed intelligence enhances scalability and resilience, effective cooperation among autonomous agents requires privacy-preserving learning mechanisms that enable collaborative model optimization and knowledge sharing without exposing sensitive network data [15,16]. Federated Learning (FL) has emerged as an attractive decentralized learning paradigm in which geographically distributed edge nodes collaboratively optimize a shared global model while retaining raw training data locally [16,17].

Recent studies have highlighted complementary advances in edge intelligence, intelligent agent communication, and distributed cybersecurity. Kong et al. [18] reviewed edge-driven IoT architectures, Zhu et al. [19] analyzed communication mechanisms for collaborative multi-agent systems, and Pawlicki et al. [5] summarized security and trust challenges across cloud–edge–IoT infrastructures. Nevertheless, these studies address computing, communication, learning, and security largely as independent research directions, leaving the integration of intelligent agent communication, federated learning, secure collaboration, and autonomous cyber defense largely unexplored. There remains a need for unified frameworks in which intelligent agents can communicate securely, exchange threat intelligence, coordinate distributed learning, and autonomously orchestrate cyber defense across edge–cloud IoT environments.

To address this gap, this paper proposes FMAD (Federated Multi-Agent Defense), a conceptual federated multi-agent framework for autonomous cyber defense in edge–cloud IoT environments. FMAD establishes an architectural framework in which specialized autonomous agents collaboratively perform traffic sensing, threat intelligence, federated model optimization, secure communication, and adaptive mitigation. To demonstrate the feasibility of the proposed framework, only the Threat Intelligence Agent (TIA) is implemented as a proof of concept using an xLSTM-based temporal intrusion detection model trained on the CIC-IoT-2023 dataset. The remaining architectural components, including FL coordination, secure communication management, and blockchain-based trust management, are not experimentally evaluated in this study. In addition, FMAD incorporates Elliptic Curve Cryptography (ECC) for lightweight key establishment, ChaCha20-Poly1305 authenticated encryption for confidential and integrity-protected communication, and a permissioned blockchain to provide trustworthy, transparent, and traceable collaboration among distributed autonomous agents.

Unlike existing studies that primarily focus on intrusion detection models or secure communication mechanisms independently, this study considers intelligent agent communication itself as the core architectural component enabling autonomous cyber defense. Within FMAD, communication extends beyond secure message transmission by allowing autonomous agents to exchange contextual threat intelligence, coordinate mitigation strategies, synchronize learning processes, and collaboratively support distributed decision-making across edge–cloud IoT environments.

The main contributions of this study are summarized as follows:

- FMAD, a conceptual federated multi-agent framework, is proposed to enable autonomous cyber defense in edge–cloud IoT environments through intelligent agent collaboration, federated learning, and secure coordination.
- A conceptual five-agent architecture is introduced to define the functional responsibilities and interactions of the Traffic Sensing Agent, Threat Intelligence Agent, Federated Learning Agent, Secure Communication Agent, and Adaptive Mitigation Agent within a unified cyber defense framework.
- As a proof of concept, the TIA is implemented using an xLSTM-based temporal intrusion detection model trained on the CIC-IoT-2023 dataset, demonstrating the feasibility of intelligent edge-based threat detection within the proposed framework.
- A design for lightweight and trustworthy inter-agent communication is presented by integrating ECC key agreement, ChaCha20-Poly1305 authenticated encryption, and a permissioned blockchain to **support** confidentiality, integrity, authentication, and traceability of distributed agent interactions.
- The proposed FMAD framework **integrates** federated learning with intelligent multi-agent collaboration as an architectural mechanism for privacy-preserving distributed cyber defense without exchanging raw IoT traffic data.
- Finally, the study distinguishes between the architectural contribution of the FMAD framework and its empirical validation. FMAD is presented as an integrated multi-agent architecture, while the experimental evaluation specifically validates the xLSTM-based TIA for DDoS detection. The remaining agents and federated-learning workflow are analyzed at the architectural, security, and operational-design levels rather than claimed as experimentally validated components.

The remainder of this paper is organized as follows. Section 2 reviews the related work on intelligent multi-agent systems, federated learning, secure communication, blockchain-based trust management, and autonomous cyber defense in edge–cloud IoT environments. Section 3 presents the theoretical foundations of the enabling technologies adopted in this work. Section 4 introduces the proposed FMAD framework and describes the architecture, functional roles, and interactions of its autonomous agents. Section 5 presents the implementation of the proof-of-concept TIA based on the proposed xLSTM model, followed by the experimental evaluation. Section 6 discusses the findings, limitations, and future research directions. Finally, Section 7 concludes the paper.

## 2. Related Work

By moving computational resources closer to data sources, edge computing significantly reduces communication latency, alleviates network congestion, and enables real-time analytics for latency-sensitive applications, thereby supporting scalable intelligent services in domains such as smart cities, industrial automation, healthcare, and intelligent transportation systems [1,4]. More recently, the emergence of Edge Intelligence has further enhanced these capabilities by integrating artificial intelligence directly into edge devices, enabling autonomous data processing and distributed decision-making while reducing dependence on centralized cloud infrastructures [2]. Among various cyber threats, Distributed Denial-of-Service (DDoS) attacks remain one of the most disruptive because compromised IoT devices can be organized into large-scale botnets capable of exhausting communication bandwidth, computational resources, and service availability [7,20].

The limitations of centralized cyber defense have motivated the development of distributed intelligent security architectures capable of operating across geographically dispersed IoT environments. Among these approaches, MAS have emerged as a promising paradigm for implementing autonomous and distributed cyber defense through cooperation among multiple intelligent agents [21,22]. Rather than relying on centralized decision-making, autonomous agents independently perceive local environmental conditions, exchange information, perform collaborative reasoning, and execute coordinated defensive actions to achieve individual and collective objectives [13,21]. To facilitate interoperability among heterogeneous agents, the Foundation for Intelligent Physical Agents (FIPA) established standardized Agent Communication Languages (ACL), interaction protocols, and agent management specifications that support agent discovery, negotiation, cooperation, and message exchange in distributed environments [23]. These standards have been implemented in widely adopted development platforms such as JADE [24], enabling practical deployment of interoperable multi-agent applications.

Recent research has further extended the role of MAS from distributed automation toward autonomous cyber defense. Kott et al. [12] introduced the concept of Autonomous Intelligent Cyber-defense Agents (AICA), demonstrating that intelligent agents can collaboratively perform sensing, threat analysis, planning, learning, and defensive actions while continuously adapting to evolving cyber attacks. Similarly, recent surveys indicate that MAS provides an effective foundation for distributed cyber defense by supporting collaborative sensing, decentralized decision-making, adaptive response, and fault-tolerant operation across dynamic computing environments [14,15].

While MAS provides the architectural foundation for distributed cyber defense, effective cooperation among autonomous agents also depends on privacy-preserving collaborative learning. In large-scale edge–cloud IoT environments, transmitting raw network traffic from distributed edge nodes to centralized servers not only incurs excessive communication overhead but also raises significant privacy, security, and regulatory concerns. To address these limitations, FL has emerged as a decentralized machine learning paradigm that enables geographically distributed nodes to collaboratively optimize a shared global model while keeping raw training data locally. Instead of exchanging sensitive traffic records, participating nodes periodically transmit only model parameters or gradients, thereby reducing communication costs and improving data privacy [16,17]. Consequently, federated learning has become one of the key enabling technologies for scalable and privacy-preserving intelligence in edge–cloud IoT systems.

Recent studies have demonstrated the effectiveness of FL for distributed intrusion detection, anomaly analysis, and cyber threat intelligence across heterogeneous IoT environments. Alongside distributed learning, DL has become one of the dominant approaches for network intrusion detection because of its ability to automatically extract discriminative features from high-dimensional traffic data. Compared with conventional ML algorithms that rely heavily on handcrafted features, deep neural networks can learn complex temporal and spatial representations directly from network traffic, resulting in improved detection accuracy for sophisticated cyber attacks [8,9]. Among these architectures, LSTM networks have demonstrated remarkable effectiveness in modeling sequential network traffic by capturing long-term temporal dependencies, making them particularly suitable for detecting DDoS attacks characterized by evolving communication patterns [25,26].

More recently, next-generation recurrent architectures such as Extended Long Short-Term Memory (xLSTM) have been proposed to overcome several limitations of conventional LSTM models, including limited memory utilization, restricted long-range dependency modeling, and reduced scalability for large-scale sequential learning tasks. By introducing enhanced memory structures and improved gating mechanisms, xLSTM provides stronger sequence modeling capability while maintaining computational efficiency, making it an attractive alternative for intelligent cyber threat detection in dynamic edge environments [27].

In parallel with advances in distributed intelligence and collaborative learning, secure communication and trust management have become indispensable components of autonomous cyber defense architectures. Elliptic Curve Cryptography (ECC) has been widely adopted for efficient public-key establishment in IoT environments because it achieves security comparable to traditional public-key cryptography while using significantly smaller key sizes [28,29]. Similarly, ChaCha20-Poly1305 has emerged as an efficient authenticated encryption scheme capable of simultaneously providing confidentiality and message integrity with low computational complexity, making it particularly suitable for constrained edge and IoT devices [30].

Beyond protecting communication channels, distributed cyber defense also requires mechanisms for establishing trust among autonomous entities operating without centralized supervision. Blockchain technology has therefore attracted considerable attention as a decentralized trust infrastructure capable of providing immutable logging, transparent collaboration, and verifiable interaction histories across distributed systems [31,32]. More recently, the adoption of Zero Trust Architecture (ZTA) has further reinforced this direction by promoting continuous verification of users, devices, and services rather than assuming inherent trust within network boundaries [33]. Collectively, these technologies provide the fundamental security primitives required for trustworthy cooperation among distributed intelligent agents. Nevertheless, most existing studies investigate cryptographic protection, blockchain-based trust management, or zero-trust principles as independent security mechanisms rather than integrating them into a unified autonomous cyber defense architecture.

To better illustrate this research landscape, Table 1 compares representative studies with respect to distributed intelligence, collaborative learning, secure communication, trust management, and autonomous cyber defense capabilities. As shown, existing approaches typically address individual components of autonomous cyber defense, whereas comprehensive integration across these complementary technologies remains limited.

Table 1 summarizes representative studies related to DDoS detection, federated learning, multi-agent security, and distributed cyber defense.

Existing studies have addressed individual aspects of autonomous cyber defense for edge–cloud IoT environments, including deep learning-based intrusion detection, federated learning, multi-agent systems, and blockchain-based trust management. However, these approaches predominantly focus on isolated research problems rather than providing an integrated architecture that enables intelligent agent communication, privacy-preserving collaborative learning, secure inter-agent communication, decentralized trust management, and autonomous cyber defense within a unified framework. Consequently, the lack of a comprehensive federated multi-agent architecture remains an important research gap. To address this gap, this study proposes FMAD, a federated multi-agent framework that integrates intelligent agent communication, federated learning, lightweight cryptography, blockchain-based trust management, and adaptive cyber defense into a unified architecture. As a proof of concept, the TIA is implemented using an xLSTM-based temporal intrusion detection model to demonstrate the feasibility of intelligent threat detection within the proposed framework.

Table 2 provides a contextual summary of the literature rather than a definitive performance ranking.

Among the studies reviewed, Hekmati et al. [34] achieved an 81% F1 score for camouflaged DDoS attacks using correlation-aware LSTM on a 4060-node IoT testbed. Jakotiya et al. [35] reported 99.84% accuracy with federated neural networks on CIC-IoT-2023, while Jony and Arnob [36] attained 98.75% accuracy using LSTM. Ain et al. [37] proposed a hybrid CNN-LSTM–Autoencoder model reaching 96.78% accuracy on the same dataset. Regarding xLSTM-based approaches, Çekiş et al. [38] compared eight deep learning models including xLSTM for IP spoofing detection, achieving near-perfect accuracy, and Baalia et al. [39] applied xLSTM variants to SCADA intrusion detection. The present study achieves 99.89% accuracy with the xLSTM-based TIA, demonstrating competitive performance while also reporting training and testing times—a critical factor often overlooked in related work, yet essential for real-time edge–cloud IoT deployments.

## 3. Theoretical Foundations

### 3.1. Edge–Cloud IoT as a Distributed Cyber Defense Environment

The convergence of the IoT, edge computing, and cloud computing has fundamentally reshaped modern distributed computing infrastructures [1,2,3]. Unlike traditional cloud-centric architectures, where sensing devices continuously transmit raw data to centralized data centers, edge–cloud computing distributes computation, storage, and intelligence across multiple hierarchical layers. This architecture enables latency-sensitive applications to process data closer to its source while exploiting the cloud for computationally intensive tasks, thereby improving responsiveness, scalability, bandwidth efficiency, and service reliability [1,4]. Protecting such environments requires cybersecurity mechanisms capable of operating collaboratively across multiple network layers while adapting to continuously changing operational conditions [11]. Among the diverse threats affecting edge–cloud infrastructures, DDoS attacks remain one of the most disruptive because they directly target service availability by exhausting communication, computational, or storage resources [20]. The proliferation of IoT botnets has significantly increased the scale of these attacks, allowing thousands of compromised devices to generate coordinated malicious traffic capable of simultaneously overwhelming edge gateways and cloud services [7]. Moreover, the distributed nature of edge–cloud architectures enables attack traffic to propagate across multiple computational layers, making early detection considerably more difficult than in conventional centralized environments. Cybersecurity solutions must continuously analyze sequential traffic behavior instead of relying solely on static signatures or predefined attack rules. This shift has accelerated the adoption of intelligent and distributed defense mechanisms capable of performing localized monitoring, collaborative threat analysis, and adaptive response across geographically distributed edge infrastructures [9].

### 3.2. Multi-Agent Systems for Distributed Cyber Defense

Unlike centralized software architectures, decision-making in a MAS is distributed among multiple autonomous agents, allowing the overall system to continue operating even when individual agents experience failures or communication disruptions [13,21]. These characteristics make MAS particularly suitable for large-scale edge–cloud IoT environments, where computational resources, sensing capabilities, and security responsibilities are inherently distributed across geographically separated nodes. Decentralized intelligence significantly improves scalability, responsiveness, and fault tolerance, enabling cybersecurity operations to remain effective despite dynamic network conditions and partial infrastructure failures [22]. The Foundation for Intelligent Physical Agents (FIPA) specifications provide standardized communication languages and interaction protocols that enable interoperable agent discovery, message exchange, negotiation, and cooperative problem solving among heterogeneous agents [23]. These standards have established the theoretical basis for secure and interoperable agent communication in distributed intelligent systems and remain widely adopted in contemporary multi-agent research. Distributed agent collaboration enhances resilience by eliminating single points of failure that frequently limit conventional centralized security architectures [12]. The coordination of autonomous agents is commonly explained through the Belief–Desire–Intention (BDI) model, which provides a cognitive framework for rational agent behavior. Although FMAD is not a strict implementation of a classical BDI architecture, its operational workflow follows similar principles by enabling individual agents to collect localized security information, collaboratively exchange threat intelligence, and coordinate adaptive cyber-defense actions according to their specialized responsibilities. Accordingly, MAS establishes the backbone of the proposed FMAD framework. Instead of treating cyber defense as a centralized monitoring task, FMAD distributes sensing, intelligence generation, secure communication, collaborative learning, and adaptive mitigation among specialized autonomous agents operating across edge–cloud infrastructures. This decentralized organization **is intended to provide** architectural flexibility and support scalability and resilience for autonomous cyber defense while also supporting integration with federated learning, lightweight cryptography, and blockchain-based trust management, which are discussed in the following sections.

## 4. Proposed FMAD Framework

### 4.1. Design of the FMAD Architecture

The FMAD framework establishes a decentralized architectural blueprint for autonomous cyber defense in edge–cloud IoT environments, integrating intelligent agent collaboration, privacy-preserving federated learning, and secure inter-agent communication into a unified operational model. Unlike centralized security architectures that introduce single points of failure and scalability limitations, FMAD distributes cyber defense responsibilities across five specialized autonomous agents operating collaboratively across hierarchical edge–cloud layers.

The architecture comprises five functionally distinct agent types:

- Traffic Sensing Agent (TSA): Deployed at edge gateways and fog nodes, the TSA performs continuous packet-level traffic acquisition and preprocessing. It extracts flow-based features from raw network traffic, applies data normalization, and prepares structured observations for downstream threat analysis. The TSA maintains local traffic profiles and communicates statistical summaries—rather than raw payloads—to preserve data locality.
- Threat Intelligence Agent (TIA): The TIA operates at the edge intelligence layer, executing temporal anomaly detection using an xLSTM-based deep learning model developed in this study. It processes preprocessed traffic features to identify DDoS patterns with 99.89% detection accuracy on the CIC-IoT-2023 dataset. The TIA maintains a local detection model and generates threat alerts upon identifying anomalous behavior, forming the cognitive core of the framework’s intrusion detection capability.
- Federated Learning Agent (FLA): The FLA orchestrates collaborative model optimization across distributed edge nodes. Instead of exchanging sensitive network data, each FLA participates in federated aggregation rounds by transmitting encrypted local model updates (gradients or weights) to a central aggregation server. This mechanism enables global model improvement while preserving data privacy and reducing communication overhead—a critical requirement for bandwidth-constrained IoT environments.
- Secure Communication Agent (SCA): The SCA implements lightweight cryptographic protection for all inter-agent interactions. It employs Elliptic Curve Cryptography (ECC) for ephemeral key agreement, ChaCha20-Poly1305 for authenticated encryption providing confidentiality, integrity, and authenticity, and TLS 1.3 for secure channel establishment. The SCA also maintains session keys and manages certificate-based authentication among participating agents.
- Adaptive Mitigation Agent (AMA): The AMA executes defensive countermeasures upon receiving threat intelligence from TIAs. Its responsibilities include traffic filtering, rate limiting, dynamic access control rule updates, and resource reallocation to preserve service availability. The AMA employs predefined mitigation policies while retaining capacity for adaptive response based on evolving attack characteristics.

**Figure 2:**
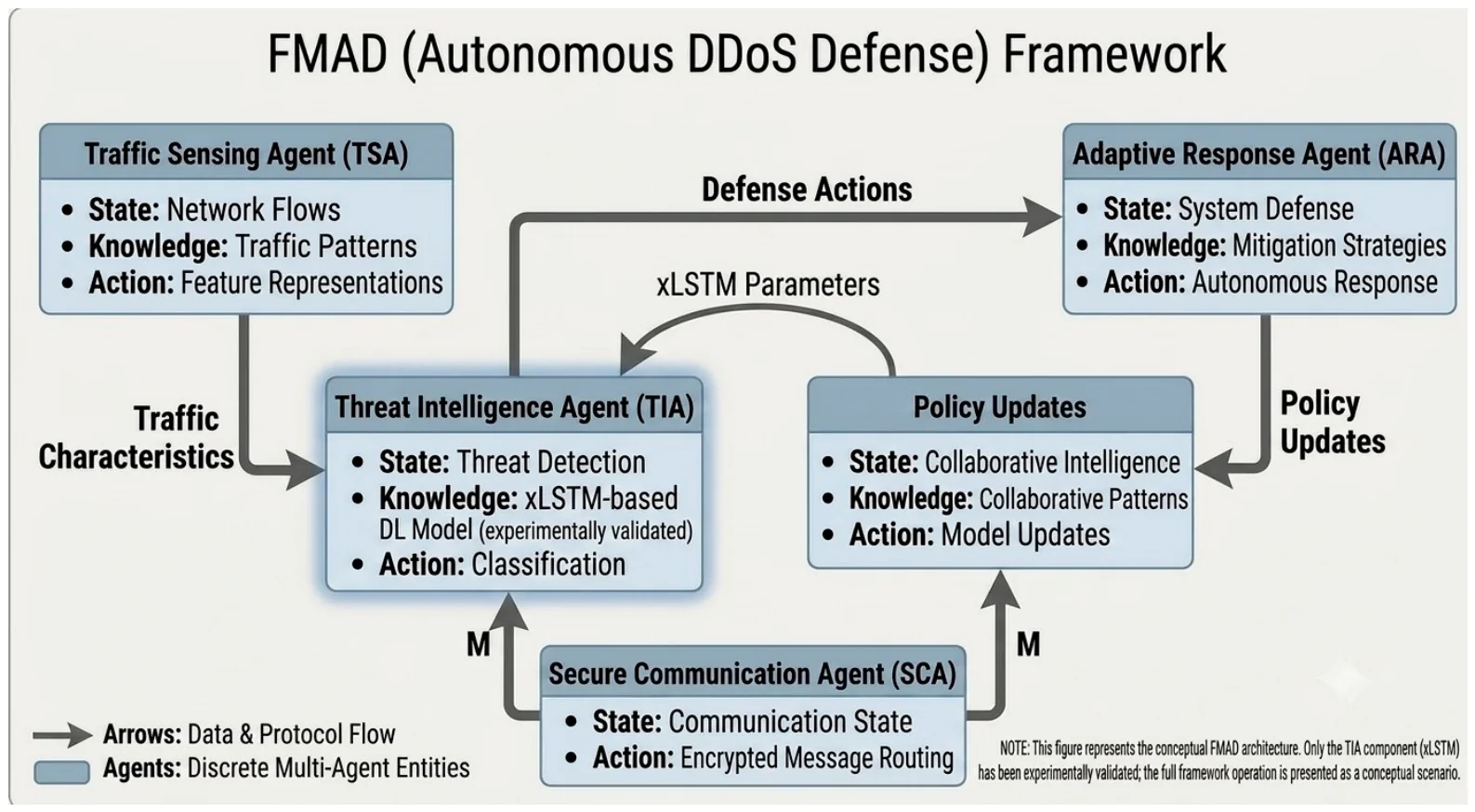
FMAD Framework Architecture for Autonomous DDoS Defense.

The FMAD architecture is shown in Figure 2.

The TSA continuously monitors network traffic at edge nodes and forwards structured traffic data to the TIA for in-depth analysis. The TIA performs xLSTM-based temporal intrusion detection to identify malicious patterns within the network traffic. When a potential threat is detected, the TIA generates threat intelligence, including classification results and confidence scores, and transmits this information to the AMA and other agents through secure channels established by the SCA. Upon receiving confirmed threat intelligence, the AMA executes autonomous defense actions, including traffic filtering, rate control, and resource adaptation, thereby enhancing system resilience against cyberattacks. Simultaneously, the FLA coordinates privacy-preserving collaborative learning by collecting local model updates from distributed edge nodes and aggregating them through federated optimization, ensuring that the global detection model continuously improves without exposing raw network data. The SCA manages all inter-agent communications through lightweight cryptography, establishing secure channels that guarantee confidentiality, integrity, authentication, and traceability of all exchanged messages. Acting as the trust and coordination layer, the permissioned blockchain ledger immutably records all agent activities, providing transparency, accountability, and forensic traceability for the entire defense process. This interaction among traffic sensing, threat intelligence, federated learning, secure communication, adaptive response, and blockchain-based trust management constitutes the core workflow of the federated multi-agent cyber defense system. Effective threat detection and mitigation require close collaboration and coordination among all five specialized agents operating across the edge–cloud IoT environment.

### 4.2. Secure Inter-Agent Communication

Secure communication among distributed autonomous agents is a fundamental requirement for trustworthy multi-agent cyber defense in edge–cloud IoT environments. In large-scale distributed systems, agents may be exposed to various cyber threats, including eavesdropping, message interception, tampering, impersonation, and replay attacks, which can occur during agent-to-agent interactions or inter-agent message exchanges across untrusted network segments. To mitigate these risks and ensure reliable collaboration, it is essential to establish a secure communication infrastructure designed to support confidentiality, integrity, authentication, and non-repudiation of all exchanged messages.

In the proposed FMAD framework, inter-agent communication is secured through a layered cryptographic architecture that combines lightweight public-key cryptography, authenticated encryption, and blockchain-based trust management. The Secure Communication Agent (SCA) is responsible for managing all security-critical communication operations, including key establishment, message encryption and decryption, integrity verification, and authentication of participating agents.

For key agreement and session establishment, the SCA employs Elliptic Curve Cryptography (ECC) using the X25519 key exchange function, which is based on the elliptic curve Diffie–Hellman (ECDH) protocol. ECC is preferred over conventional public-key cryptosystems such as RSA due to its significantly smaller key sizes, reduced computational overhead, and lower energy consumption, making it a suitable candidate for resource-constrained edge and IoT devices while providing comparable security strength. Through ECDH, pairs of communicating agents establish shared session keys without requiring a trusted third party, enabling secure ephemeral key exchange even in dynamically changing network topologies.

To protect the actual message payloads, the SCA utilizes ChaCha20-Poly1305, an authenticated encryption with associated data (AEAD) algorithm that simultaneously provides confidentiality, integrity, and authenticity. ChaCha20 is a stream cipher that offers high-speed encryption with constant-time execution, while Poly1305 provides message authentication through a polynomial-based message authentication code (MAC), which is used in conjunction with ChaCha20 to simultaneously provide confidentiality and integrity protection. This combination ensures that messages are not only encrypted to prevent unauthorized disclosure but also authenticated to detect any unauthorized modifications or forgery attempts during transmission. ChaCha20-Poly1305 offers improved resistance to timing side-channel attacks when implemented in constant time, and is particularly suitable for heterogeneous edge–cloud environments where AES hardware acceleration may not be available. The SCA manages session keys securely and ensures that each message is encrypted with a unique nonce to prevent replay attacks. In addition to cryptographic protection, each exchanged message carries semantic information describing its operational purpose. Threat alerts, model synchronization requests, mitigation commands, and policy updates are therefore interpreted according to their communication intention, allowing autonomous agents to coordinate distributed cyber-defense activities more effectively. Although the proposed communication mechanism is designed within FMAD, the framework assumes a standardized agent communication model inspired by the FIPA Agent Communication Language (ACL). Each inter-agent message is represented as a structured message containing fields such as performative (e.g., INFORM, REQUEST, CONFIRM, ALERT), sender identifier, receiver identifier, timestamp, message type, payload, and digital signature. This semantic message structure enables autonomous agents to exchange threat intelligence, mitigation commands, federated model updates, and coordination requests in a consistent and interoperable manner while remaining independent of any specific implementation platform.

Beyond cryptographic protection of communication channels, the FMAD framework integrates a permissioned blockchain ledger to enhance trust, transparency, and accountability in agent interactions. The blockchain layer is proposed to provide trust, traceability, and accountability among autonomous agents. However, blockchain deployment and performance evaluation are outside the scope of the current study. The blockchain is proposed as a permissioned, tamper-evident repository intended to immutably record all critical agent activities, providing traceability and auditability under the assumption that the blockchain infrastructure remains secure, including threat detection events, model update transmissions, defense actions, and system coordination decisions. Each agent’s operations are logged onto the blockchain with cryptographic signatures, providing a transparent and verifiable audit trail that cannot be altered or deleted by unauthorized actors. The Traffic Sensing Agent (TSA) utilizes the blockchain to record traffic statistics and sensing operations, ensuring that data provenance is preserved for forensic analysis. The Threat Intelligence Agent (TIA) logs detection results, including attack classifications and confidence scores, providing an immutable record of threat intelligence generation. The Federated Learning Agent (FLA) records model update transmissions and aggregation rounds, enabling transparent tracking of the collaborative learning process. The Adaptive Mitigation Agent (AMA) logs all mitigation actions, including traffic filtering rules and resource allocation decisions, ensuring that defensive measures are verifiable and accountable.

When two agents need to communicate, the SCA first establishes a secure session through ECDH key exchange. The sender agent then encrypts the message payload using ChaCha20-Poly1305 with the established session key, ensuring confidentiality and integrity. The encrypted message is transmitted over secure communication channels, and upon receipt, the receiver agent decrypts the message and verifies its authenticity using the same session key. Simultaneously, both sender and receiver log the communication event—including message metadata, timestamps, and cryptographic hashes—onto the permissioned blockchain, creating an immutable and auditable record of the interaction. The receiver additionally verifies the message authenticity by cross-referencing the corresponding blockchain entry, supporting verification of the sender’s authenticated identity and the integrity of the recorded communication event.

This comprehensive security approach—combining ECC-based key agreement, ChaCha20-Poly1305 authenticated encryption, and blockchain-based trust management—provides a designed framework intended to support secure agent-to-agent interactions, subject to the residual risks and assumptions outlined in the security model. Through this layered architecture, the framework effectively mitigates the risks associated with insecure communications in distributed edge–cloud IoT environments, providing a secure, reliable, and accountable platform for autonomous multi-agent cyber defense. Although the proposed communication architecture is designed using well-established cryptographic mechanisms, it is presented as a design within the FMAD framework. Therefore, quantitative performance metrics such as communication latency, encryption overhead, throughput, computational cost, and energy consumption are intentionally left for future experimental validation in a fully distributed FMAD deployment.

#### 4.2.1. Security Threat Model and Assumptions

The security model of FMAD distinguishes between trusted infrastructure components, authorized but potentially compromised agents, and external adversaries. In this context, the SCA and permissioned blockchain are treated as security-supporting components responsible for authenticated communication, cryptographic protection, and activity traceability. Registered agents are assumed to possess valid cryptographic credentials before participating in the system. However, participating edge agents are not assumed to be permanently trustworthy. In particular, TSA, TIA, and FLA components may be compromised by an adversary. The threat model therefore considers message interception, modification, replay, impersonation, unauthorized model-update submission, and malicious manipulation of federated learning updates as relevant attack scenarios. The FLA requires particular consideration because it currently acts as the central aggregation coordinator for federated learning. If the FLA is compromised, an attacker may manipulate the aggregation process or influence the resulting global model. Therefore, authenticated and encrypted communication protects model updates during transmission but does not by itself guarantee the correctness of updates generated by compromised participants or the integrity of an aggregation process performed by a compromised coordinator. Accordingly, the FMAD security model follows a layered approach in which cryptographic mechanisms protect communication, blockchain-based logging provides traceability and accountability, and the federated aggregation mechanism is treated as a replaceable component whose robustness can be strengthened against malicious participants. In the current design, FedAvg is retained as the baseline aggregation method, while Byzantine-resilient aggregation methods are considered as a design extension for adversarial federated learning scenarios. However, the robustness of aggregation against malicious participants was not experimentally evaluated in this study.

**Figure 3:**
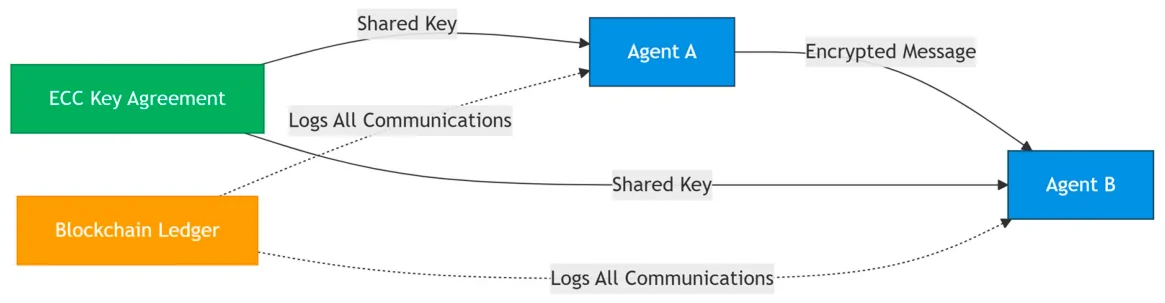
Secure inter-agent communication in FMAD framework.

As shown in Figure 3, when a DDoS attack occurs in the edge–cloud IoT environment, the secure inter-agent communication mechanism ensures reliable and trustworthy coordination among distributed autonomous agents throughout the entire defense lifecycle.

Upon detection of anomalous traffic patterns, the TSA securely transmits preprocessed traffic data to the TIA through an encrypted channel established by the SCA. The TIA, after confirming the attack using the xLSTM-based detection model, generates threat intelligence and disseminates it to the AMA through authenticated and integrity-protected messages. Simultaneously, the FLA securely exchanges encrypted model updates with distributed nodes, enabling collaborative learning without exposing sensitive network data. Throughout this coordinated defense process, the SCA is designed to provide encrypted and authenticated communication, while relevant interactions are recorded on the blockchain ledger. This secure communication infrastructure is designed to mitigate the risks of message interception and tampering with critical defense messages but also provides a transparent and auditable trail of all agent interactions, enabling forensic analysis and ensuring accountability across the entire distributed cyber defense system.

#### 4.2.2. Security Property Analysis

The principal security properties of the FMAD framework and the mechanisms supporting them are summarized in Table 3. The table also highlights the main limitations and residual risks associated with each security mechanism.

The analysis demonstrates that no individual mechanism provides complete protection against all considered threats. Cryptographic mechanisms primarily protect communication, whereas blockchain provides traceability and accountability. Federated aggregation addresses the learning layer and therefore requires separate consideration when participating clients or the aggregation coordinator may be compromised. The identified limitations are therefore treated as residual risks rather than as assumptions that the proposed mechanisms provide complete security.

### 4.3. Federated Learning (FL) for Privacy-Preserving Collaborative Defense

FL is proposed as a fundamental component of the FMAD framework, intended to support privacy-preserving collaborative intelligence across distributed edge nodes without requiring the exchange of raw network traffic data. However, the complete federated workflow was not experimentally validated in this study. In conventional centralized learning approaches, aggregating sensitive IoT traffic data from geographically dispersed edge nodes to a central server raises significant privacy concerns, induces excessive communication overhead, and creates potential single points of failure. To address these limitations, the FMAD framework integrates FL as a decentralized learning paradigm in which multiple edge nodes collaboratively train a shared global detection model while maintaining their local training data on-device.

The FL mechanism in FMAD operates through the coordinated efforts of the FLA and distributed TIAs deployed at edge nodes. Each TIA maintains a local xLSTM-based intrusion detection model trained on locally captured network traffic data. Instead of transmitting raw traffic payloads, each TIA periodically computes local model updates—specifically, gradients or weight parameters—based on its local data distribution. These encrypted model updates are securely transmitted to the FLA through communication channels established by the Secure Communication Agent (SCA), ensuring confidentiality and integrity during transmission.

The FLA serves as the central aggregation coordinator, implementing the Federated Averaging (FedAvg) algorithm to aggregate incoming local model updates from participating edge nodes. The aggregation process computes a weighted average of the local model parameters, producing an updated global model that encapsulates collective knowledge learned across the distributed edge infrastructure. The FLA then securely distributes the aggregated global model back to the participating TIAs, enabling each edge node to benefit from the collective intelligence of the entire network without exposing its local data. This iterative process of local training, encrypted parameter exchange, secure aggregation, and global model distribution enables continuous model improvement across the distributed system while strictly preserving data privacy. Unlike conventional federated learning architectures where communication occurs only between clients and the aggregation server, FMAD embeds federated learning into a broader intelligent agent communication ecosystem in which autonomous agents collaboratively exchange contextual security knowledge while preserving data privacy.

The integration of FL within FMAD provides several critical advantages for autonomous cyber defense in edge–cloud IoT environments. First, it may reduce communication overhead by transmitting only compact model parameters rather than voluminous raw network traffic data, which is particularly beneficial for bandwidth-constrained edge environments. Second, it supports data-locality and privacy-preserving learning by keeping sensitive IoT data localized, addressing growing concerns regarding data protection and privacy regulations. Third, collaborative training may improve model robustness and generalizability by exposing the global model to diverse traffic patterns across heterogeneous edge deployments, improving detection performance for previously unseen attack variants. Fourth, distributed learning may reduce dependence on a single data source, although its resilience to node failures is not experimentally evaluated in the current study.

**Figure 4:**
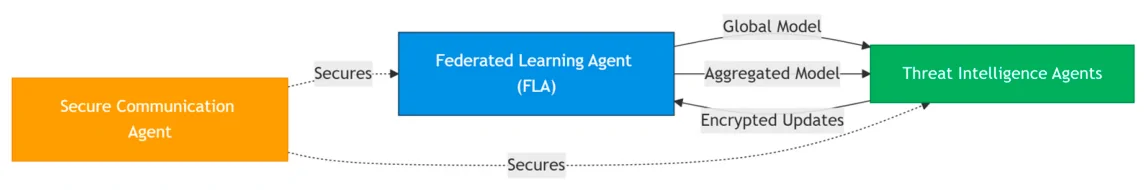
Federated learning process in FMAD framework.

The FL process is illustrated in Figure 4.

At each federated round, the FLA distributes the current global model to participating TIAs. Each TIA performs local training on its private edge traffic data, computes model updates, and securely transmits encrypted updates to the FLA via the SCA. The FLA aggregates received updates using the FedAvg algorithm, producing an improved global model that is then redistributed to all participating TIAs for the next training round. This iterative collaborative learning mechanism is intended to support continuous adaptation to evolving attack patterns while preserving data locality and potentially reducing communication overhead. However, formal privacy guarantees are not established in this study, and the end-to-end federated learning performance was not experimentally evaluated. In the current version of FMAD no federated training rounds, distributed model aggregation, or communication-efficient optimization experiments were executed in the current study.

Although FedAvg is used as the baseline aggregation method in the current FMAD design, the architecture is not inherently restricted to federated averaging. The FLA is defined as an aggregation coordinator, allowing the aggregation algorithm to be replaced without changing the functional roles of the other FMAD agents. FedAvg provides a simple baseline for collaborative optimization; however, heterogeneous edge devices and non-identically distributed local data may lead to differences in local model updates. FedProx can therefore be considered as an alternative when device and data heterogeneity becomes significant. In addition, coordinate-wise median, trimmed mean, and Krum can be considered when protection against malicious or Byzantine model updates is required. Thus, the proposed FMAD architecture supports a modular aggregation layer in which FedAvg can serve as the baseline, FedProx can address heterogeneous participation, and Byzantine-resilient aggregation methods can be investigated for adversarial environments. The current study retains FedAvg to establish a clear baseline, while comparative evaluation of alternative aggregation strategies is left for future distributed experiments.

### 4.4. A Scenario for FMAD Operation

**Figure 5:**
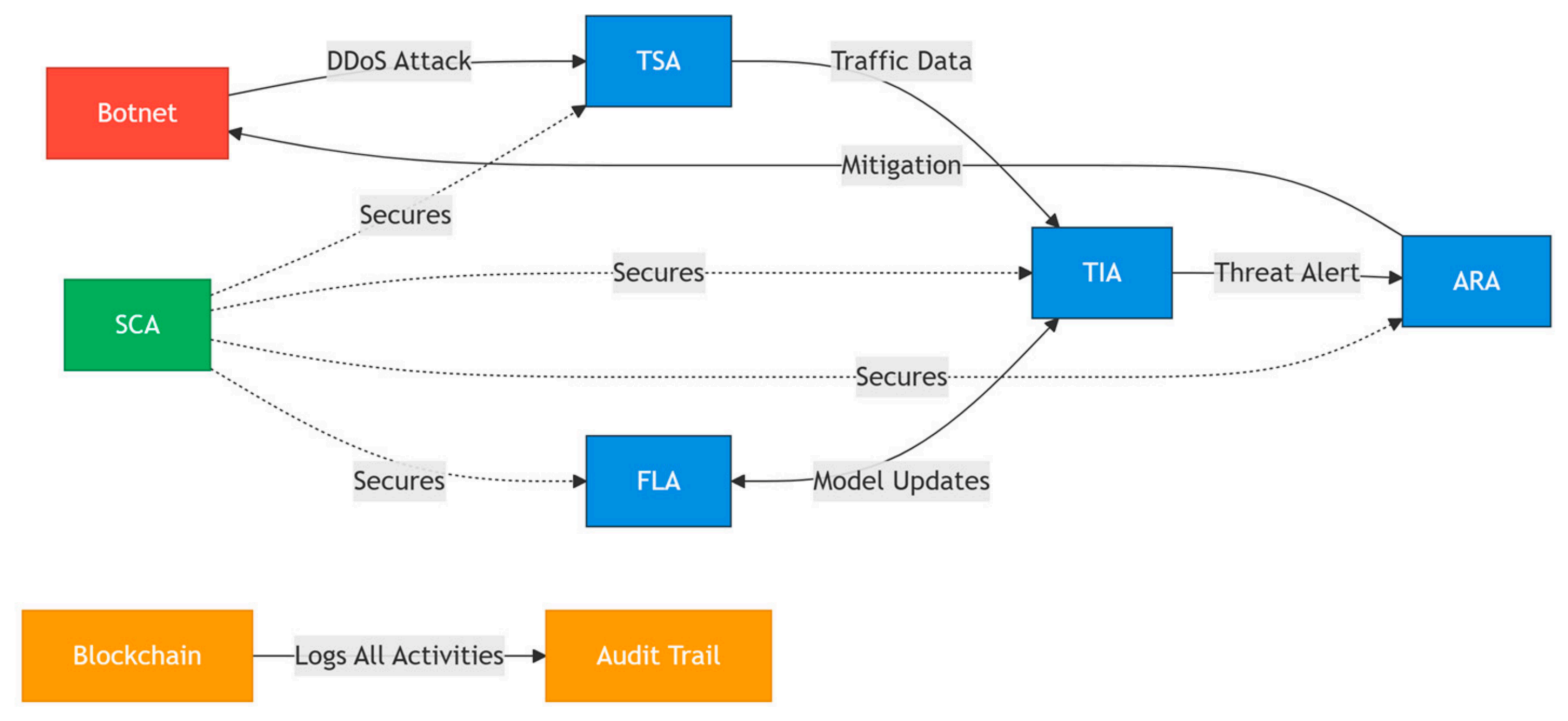
Operational scenario of the FMAD framework against DDoS attacks.

Figure 5 presents a conceptual operational scenario illustrating the intended collaborative defense mechanism of the FMAD framework against a DDoS attack in an edge–cloud IoT environment. The scenario describes how the proposed agents are designed to interact during a DDoS response, rather than reporting results from an experimentally validated deployment.

The scenario involves a large-scale botnet comprising numerous compromised IoT devices (Bot_1_, Bot_2_, …, Bot_n_) that initiate a coordinated DDoS attack targeting critical edge and cloud services. In response to this imminent threat, the FMAD framework, equipped with five specialized autonomous agents, immediately initiates its defense protocol. Each agent has a distinct role, and they work together to create a structured and resilient defense mechanism against the attack. TSAs, deployed at distributed edge nodes, continuously monitor network flows and swiftly detect anomalous traffic patterns characterized by sudden increases and unusual flow characteristics. Upon detecting signs of an attack, the TSAs immediately forward preprocessed traffic data to the TIAs through secure communication channels established by the SCAs. The TIAs, utilizing the xLSTM-based temporal intrusion detection model developed in this study, analyze the traffic patterns and confirm the attack with 99.89% detection accuracy, generating comprehensive threat intelligence including classification results and confidence scores. Upon attack confirmation, the TIAs disseminate threat intelligence to the AMAs through authenticated and integrity-protected messages. The AMAs subsequently execute autonomous defense actions, including traffic filtering, rate limiting, dynamic access control rule updates, and resource reallocation, to mitigate the attack and preserve service availability. Simultaneously, the FLAs coordinate privacy-preserving model updates across distributed nodes, enabling continuous improvement of the detection model without exposing sensitive network data. The SCAs ensure that every inter-agent communication—including threat alerts, mitigation commands, and model updates—is encrypted, authenticated, and protected against interception or tampering. Acting as the trust and coordination layer, the permissioned blockchain ledger immutably records all agent activities, including threat detection events, defense actions, and model update transmissions, providing transparent auditability and forensic traceability. In summary, the TSAs detect anomalous traffic and securely transmit data to the TIAs for attack confirmation. The TIAs generate threat intelligence and disseminate it to the AMAs, which execute autonomous mitigation measures. The FLAs coordinate collaborative model updates, while the SCAs secure all communications and the blockchain ledger ensures transparency and accountability. This coordinated multi-agent workflow represents the intended operational design of the FMAD framework for autonomous DDoS defense in edge–cloud IoT environments. It describes the proposed interactions among agents, rather than constituting an experimentally validated end-to-end deployment. It describes how the proposed agents are intended to interact and coordinate during a DDoS response, rather than constituting an experimentally validated end-to-end deployment.

## 5. Experimental Evaluation

### 5.1. Experimental Scope and Computing Environment

The experimental evaluation focuses on the xLSTM-based TIA, which represents the machine-learning component of the proposed FMAD architecture. The primary objective is to assess the capability of the TIA to distinguish DDoS-related network traffic from benign traffic. Accordingly, the quantitative results reported in this section concern the classification performance of the TIA and should not be interpreted as an end-to-end evaluation of the complete FMAD architecture.

The remaining FMAD components, including the TSA, SCA, FLA, and AMA, constitute architectural elements of the proposed framework and are described at the design level. These components were not implemented or experimentally evaluated through an end-to-end deployment in the present study. Similarly, the complete federated-learning workflow, including distributed local training, model-update exchange, aggregation, and repeated synchronization among participating nodes, was not experimentally executed. Therefore, the experimental findings are confined to the TIA component under the specified conditions. Therefore, the findings presented in this section provide empirical evidence for the TIA component under the specified experimental conditions rather than experimental validation of all FMAD capabilities.

The experiments were conducted in a cloud-based Google Colaboratory environment. TensorFlow 2.12 and Keras 2.12 were used for model implementation and training, while Python, Pandas, and NumPy were employed for data preparation and numerical processing. Computational accelerator resources available in the experimental environment were used according to the requirements of the corresponding processing stages. The overall workflow consisted of dataset preparation, feature processing, model training, validation, and independent testing.

### 5.2. Dataset Preparation and Experimental Protocol

The CIC-IoT-2023 dataset was employed to evaluate the DDoS detection capability of the proposed TIA. The dataset contains network traffic generated in IoT environments and includes both benign and malicious observations. For the binary classification task considered in this study, the analysis was restricted to the traffic categories required to distinguish DDoS-related observations from normal network activity. Prior to model development, the selected observations were shuffled and partitioned into training, validation, and test subsets using a 70%, 15%, and 15% split, respectively. The training subset was used for model parameter optimization, whereas the validation subset supported monitoring of the learning process and selection of model configurations. The test subset was kept separate from the model-development process and was reserved for the final performance assessment. This protocol was adopted to maintain a distinction between model optimization and final evaluation. The preprocessing stage included label transformation and data consistency checks. Benign traffic was assigned the binary label 0, whereas DDoS-related traffic was assigned the label 1. The input representation incorporated network-flow characteristics including bits per second, packets per second, source and destination IP addresses, source and destination ports, flow duration, and average transmitted-packet length.

**Figure 6:**
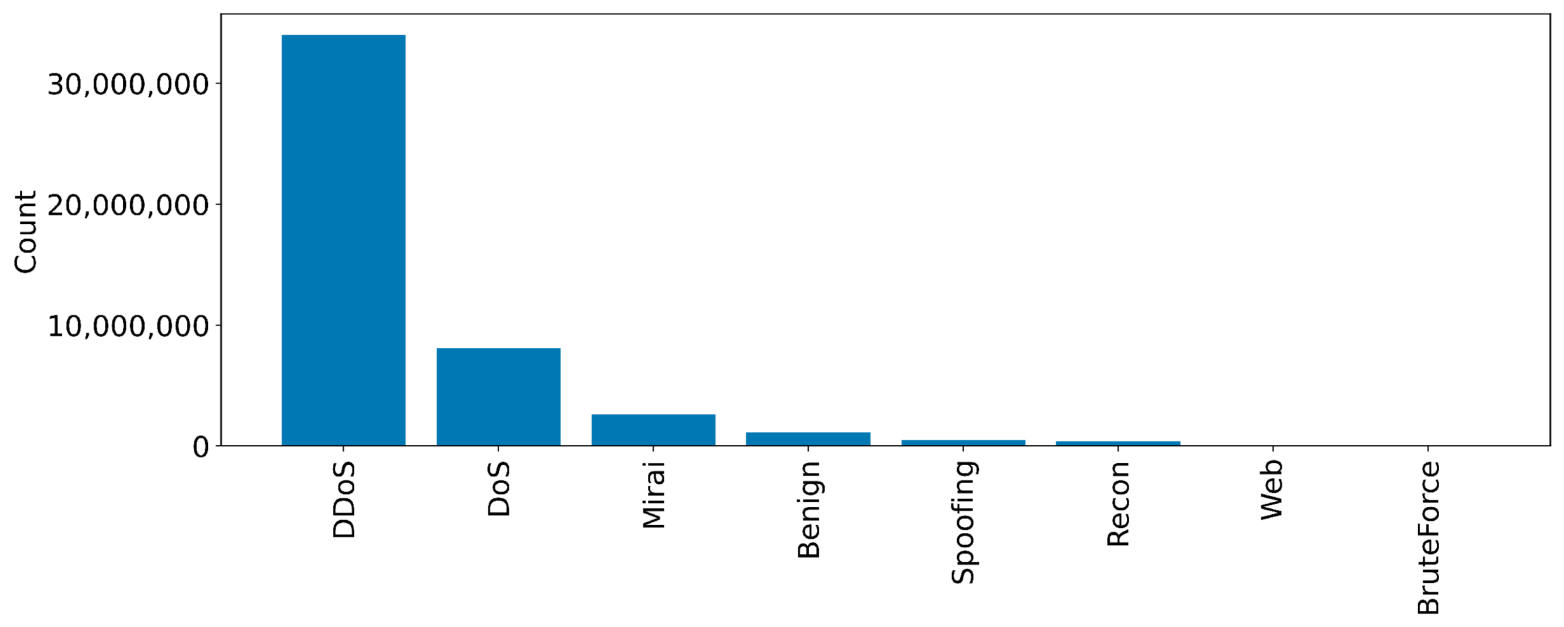
Distribution of benign and DDoS traffic in the CIC-IoT-2023 dataset.

Figure 6 illustrates the distribution of benign and DDoS-related observations considered in the experimental data.

The three subsets were used for clearly separated purposes throughout the experimental procedure. Model parameters were updated using the training data, while the validation data were used to monitor model behavior and support configuration selection. The independent test data were retained for the final assessment. This separation reduces the likelihood of information transfer between model development and performance evaluation.

### 5.3. Model Configurations and Experimental Results

Nine configurations of the xLSTM-based TIA were evaluated by varying the number of hidden layers and training epochs. This experimental design was used to examine the effect of architectural depth and training duration on the observed classification performance. RMSE, accuracy, and loss were recorded for each configuration, and the resulting measurements are presented in Table 4.

The results demonstrate that the evaluated combinations of model depth and training duration produced substantially different classification outcomes. Experiment No. 8, consisting of six hidden layers and 15 epochs, yielded the highest reported accuracy of 99.89%, together with an RMSE of 0.108 and a reported loss of 0.40%. Experiment 9 used the same number of hidden layers but extended the training period to 60 epochs. Although its RMSE decreased to 0.080, its classification accuracy was slightly lower at 99.43%.

The results also indicate that increasing the number of training epochs does not necessarily produce a monotonic increase in classification accuracy. For example, Experiment 3 reached 87.66% accuracy with two hidden layers and 60 epochs, whereas Experiment 6 achieved 97.71% accuracy using four hidden layers and the same number of epochs. Thus, the observed results suggest that model depth and training duration should be considered jointly rather than independently when selecting the TIA configuration.

**Figure 7:**
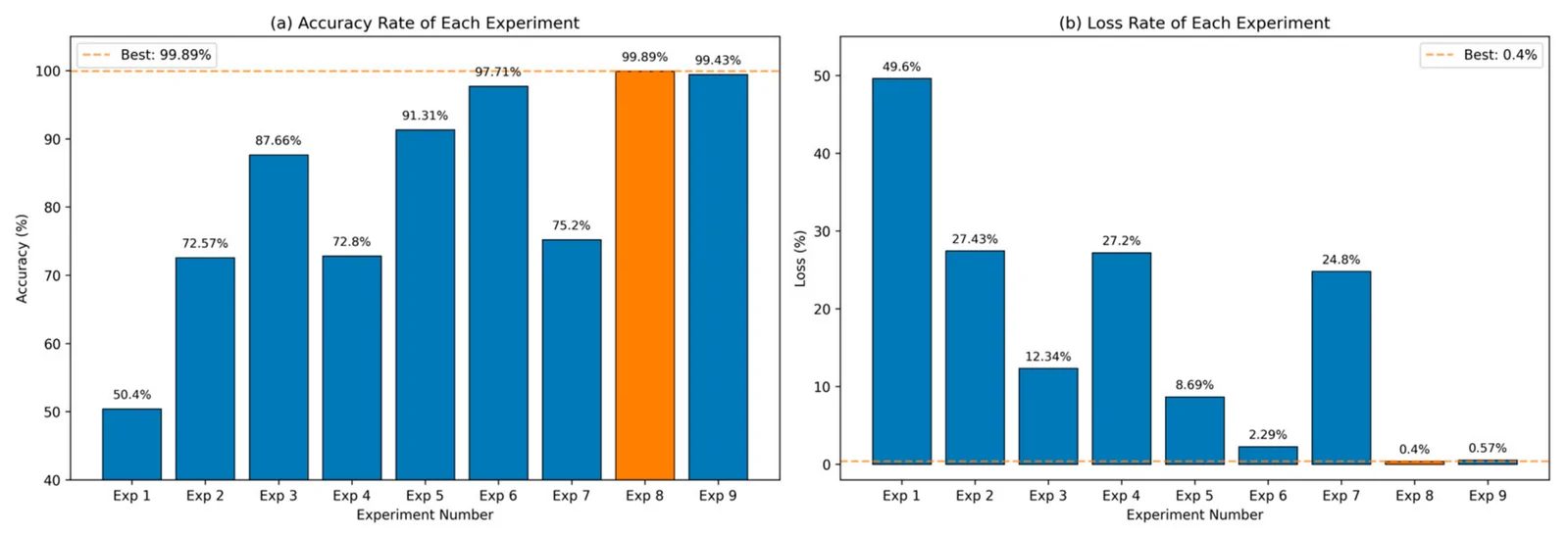
Accuracy and loss obtained for Experiment No. 8.

Experiment 8 was selected as the reference configuration for the subsequent analyses because it achieved the highest reported accuracy among the nine evaluated configurations. Its accuracy and loss values are presented in Figure 7.

**Figure 8:**
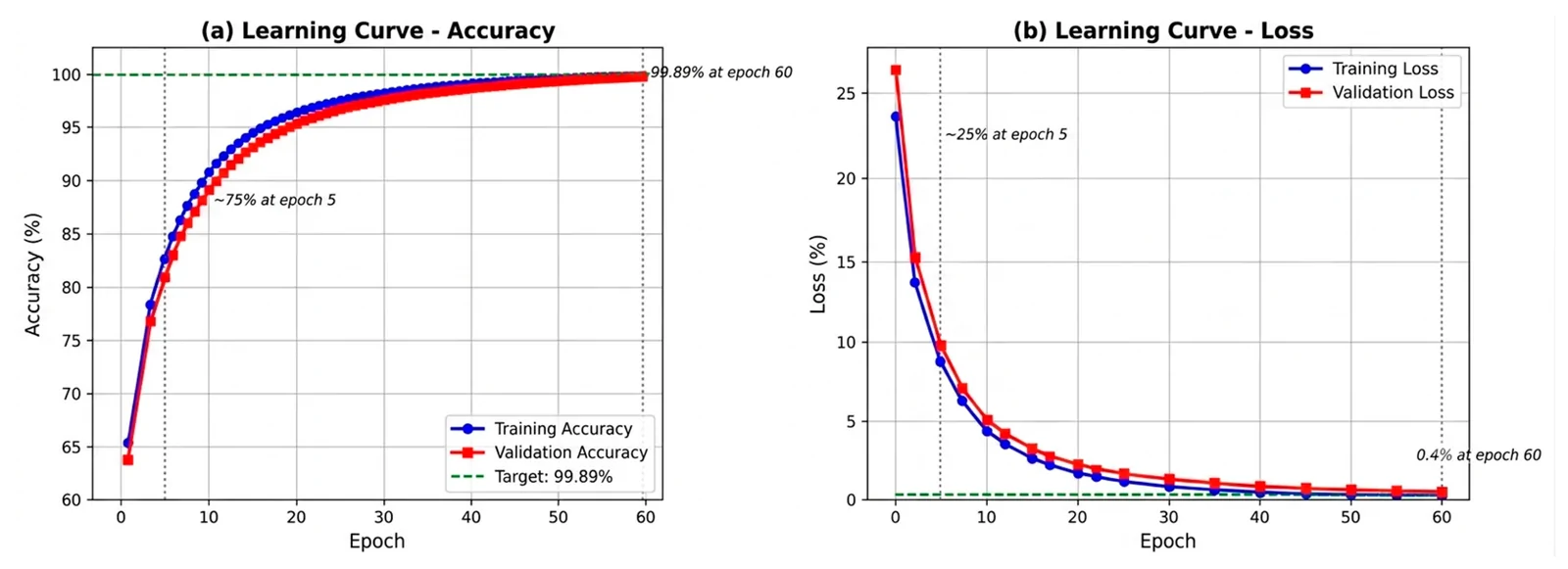
The learning curve for Experiment No. 8.

Figure 8 presents the learning curves associated with Experiment 8. The accuracy increased during training, while the loss decreased as optimization progressed. The resulting curves indicate stable learning behavior under the selected configuration. However, the experimental procedure did not include repeated independent runs with different random seeds. Therefore, the reported values represent the performance obtained under the specified experimental run rather than an estimate of variability across repeated trials.

### 5.4. Classification Performance Analysis

**Figure 9:**
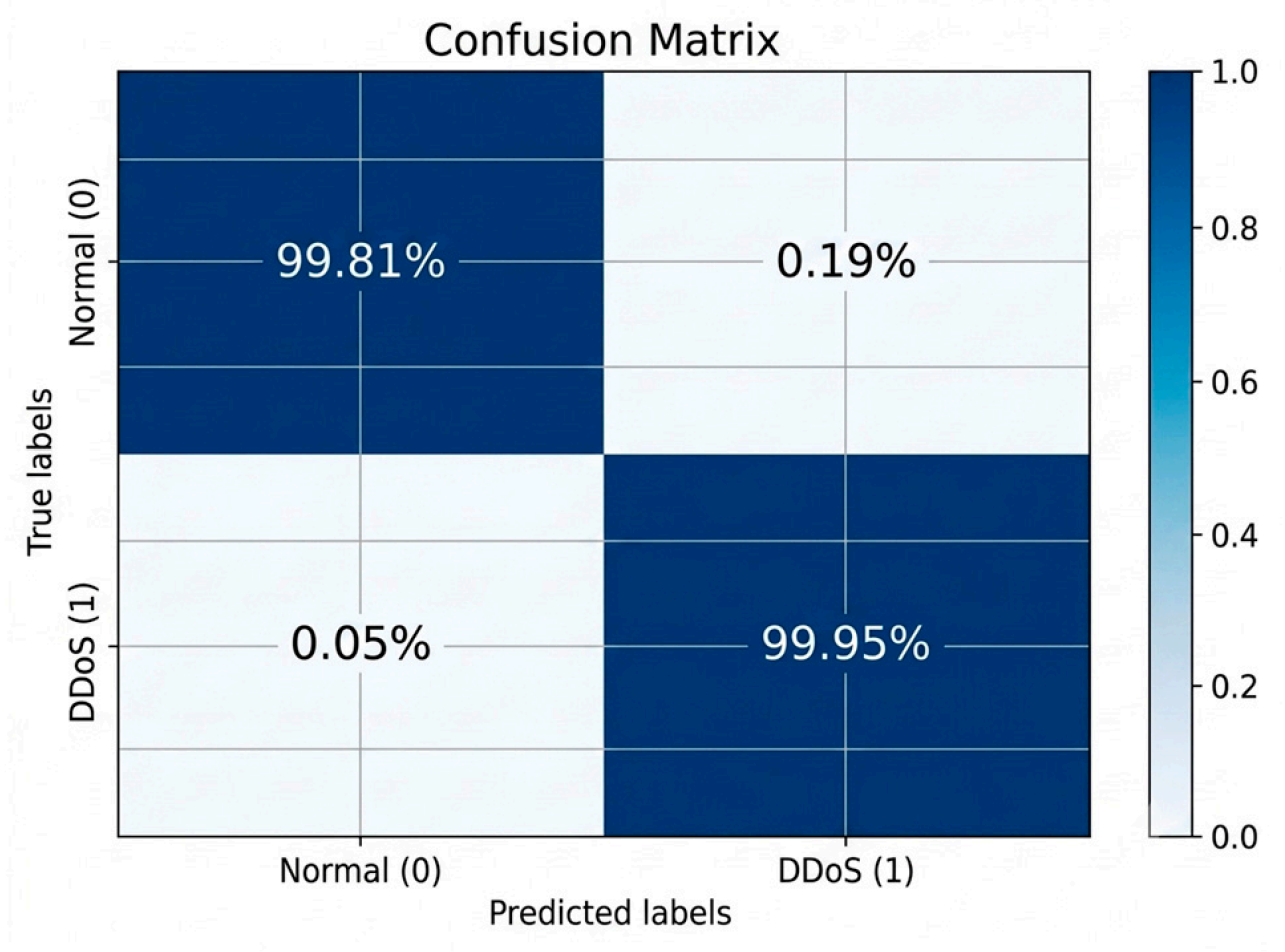
Confusion Matrix of Experiment 8.

Overall accuracy provides only a partial view of binary intrusion-detection performance. Therefore, additional classification measures were examined for Experiment 8, including precision, recall, F1-score, false positive rate (FPR), and false negative rate (FNR). The selected configuration achieved a reported precision of 99.80%, recall of 99.95%, and F1-score of 99.87%. The corresponding FPR and FNR were 0.19% and 0.05%, respectively. These measurements indicate that the classifier maintained high classification performance for both DDoS-related and benign observations within the evaluated test data. The FNR represents the proportion of attack observations incorrectly classified as benign. The reported FNR of 0.05% therefore indicates a low rate of missed attack observations in the evaluated dataset. Conversely, the FPR represents the proportion of benign observations classified as malicious; the reported value of 0.19% indicates a low false-alarm rate under the same experimental conditions. Considering these measures together provides a more informative characterization of the classifier than accuracy alone. The confusion matrix obtained for Experiment No. 8 is presented in Figure 9.

The confusion-matrix analysis yielded a True Positive Rate (TPR) of 99.95% and a True Negative Rate (TNR) of 99.81%. The corresponding FPR and FNR were 0.19% and 0.05%, respectively. These values provide additional information regarding the distribution of correct and incorrect classifications underlying the reported performance measures.

### 5.5. ROC-AUC Analysis

**Figure 10:**
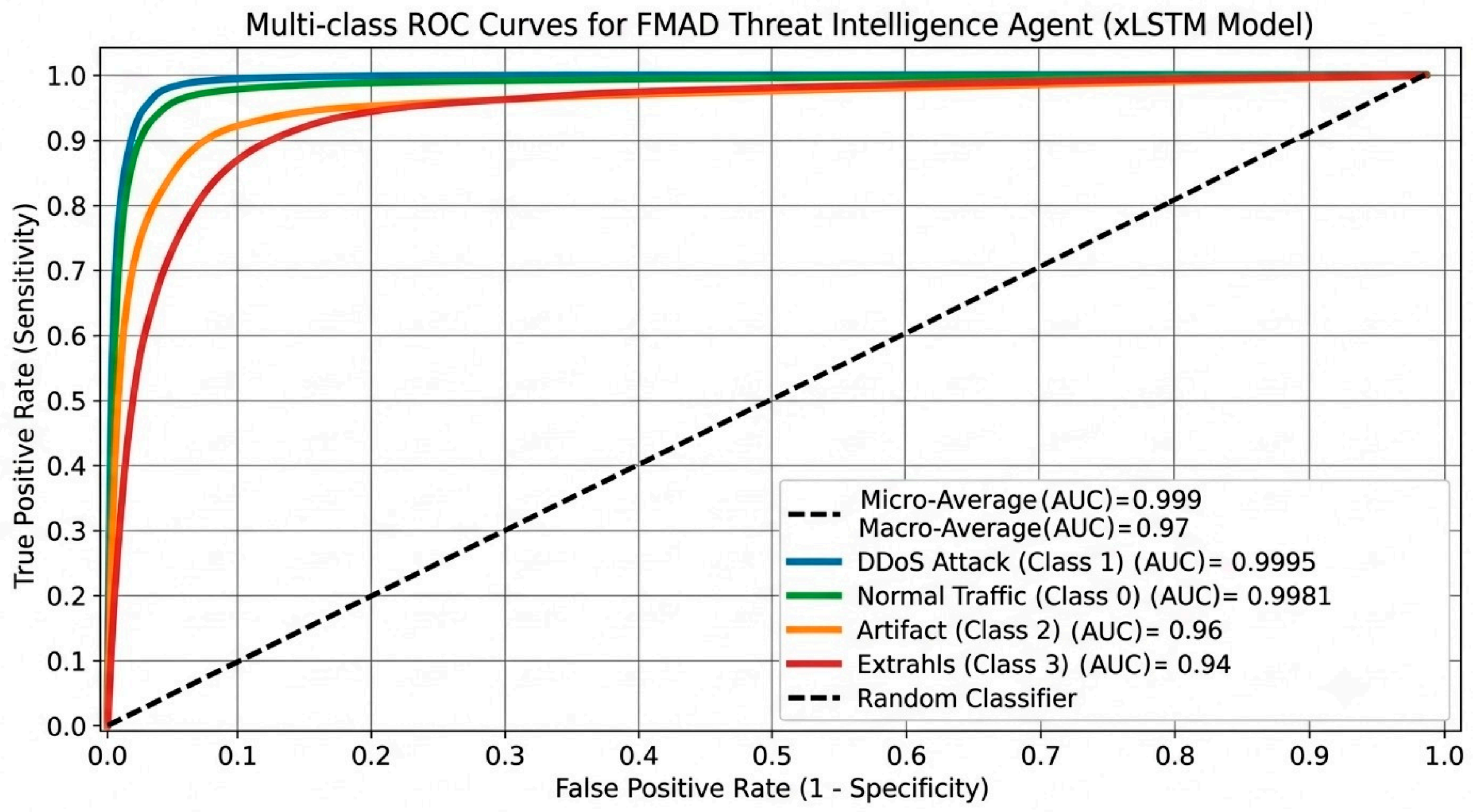
The ROC curve analysis of Experiment No. 8.

Receiver Operating Characteristic (ROC) analysis was performed to further examine the discriminatory behavior of the selected classifier across different decision thresholds. The corresponding results are shown in Figure 10.

The reported AUC values were 0.9995 for the DDoS attack class (Class 1) and 0.9981 for the benign traffic class (Class 0), while the reported micro-average AUC was 0.999. These results indicate strong discrimination between the evaluated traffic classes under the experimental conditions considered in this study.

Nevertheless, the reported AUC values should be interpreted within the scope of the selected dataset and experimental protocol. High performance on a benchmark dataset does not necessarily imply equivalent performance under different network conditions, previously unseen IoT environments, or operational deployment scenarios. Evaluation using independent datasets, cross-environment testing, and unseen traffic sources would therefore be required to assess broader generalization.

**Figure 11:**
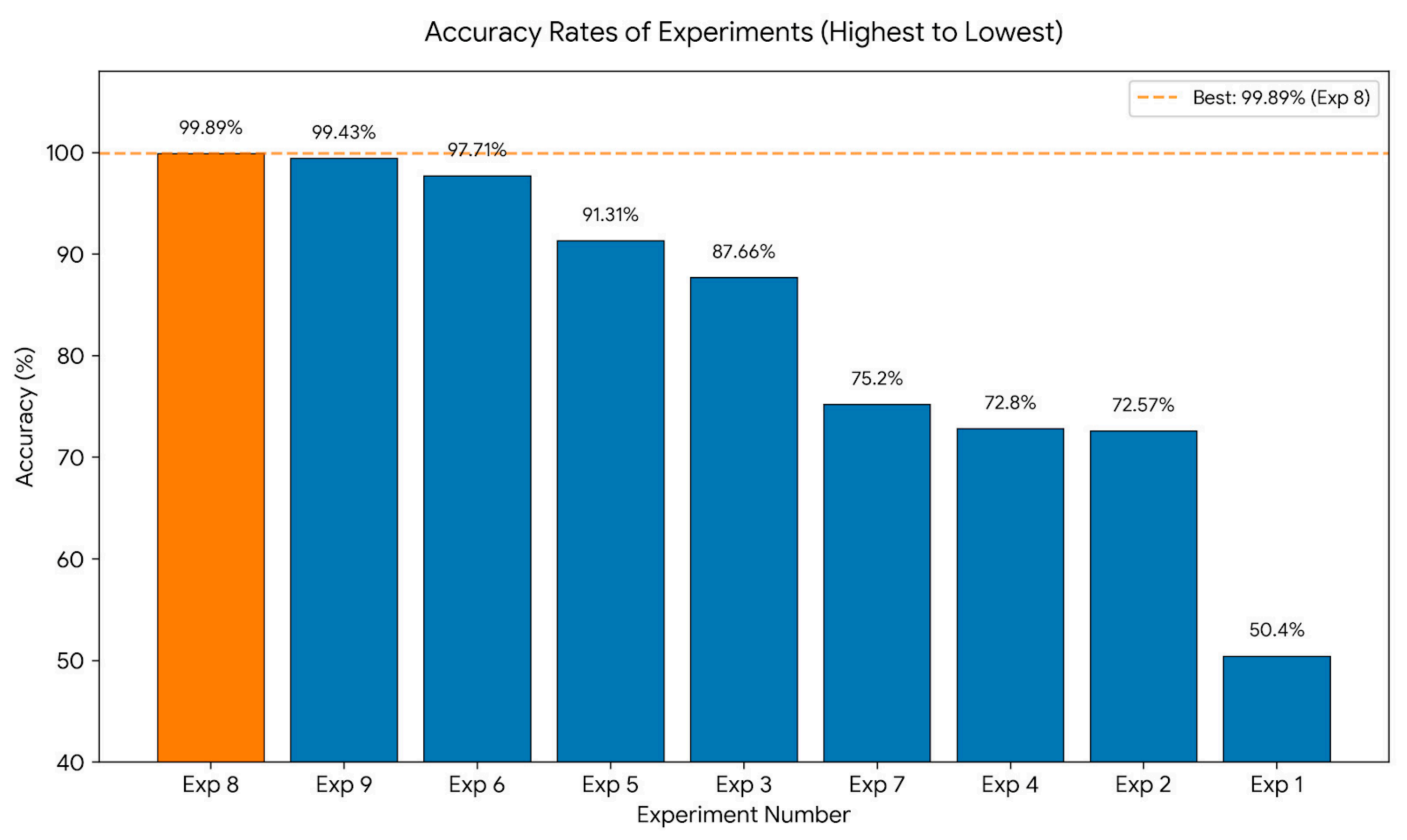
The accuracy results obtained from the experiments.

The accuracy values obtained from the nine experimental configurations are summarized in Figure 11.

### 5.6. Computational Considerations

Computational efficiency is particularly relevant to FMAD because the proposed architecture is intended for edge–cloud IoT environments, where processing capacity, memory, communication bandwidth, and energy availability may be constrained. In a complete deployment, the computational burden would include model inference as well as local training, cryptographic processing, model-update transmission, and federated aggregation.

The current experiments directly assess the computational behavior of the xLSTM-based TIA but do not experimentally execute the complete distributed FMAD workflow. Distributed local training, federated aggregation, repeated synchronization, and inter-node communication were not measured as part of the present evaluation. Consequently, the study does not provide an end-to-end measurement of energy consumption or communication overhead for the complete FMAD architecture.

A comprehensive computational evaluation of FMAD would require additional measurements, including local training duration, inference latency, memory consumption, processor utilization, model size, communication volume per federated round, aggregation time, and energy consumption. Such measurements should preferably be obtained on representative edge devices rather than exclusively on cloud-based computing resources. Future experiments should also investigate whether reducing local training epochs, modifying synchronization frequency, or adopting communication-efficient model-update strategies can reduce resource requirements while maintaining acceptable detection performance.

### 5.7. Complexity Considerations

The computational complexity of the xLSTM-based TIA depends on several factors, including the network architecture, number of trainable parameters, input sequence length, and implementation characteristics. Consequently, the complete model should not be assigned a single constant-order complexity without specifying these factors.

Let *P* denote the number of trainable parameters and (T) denote the input sequence length. The computational requirements of processing an input sequence depend on the operations performed across the model parameters and sequence positions. The exact asymptotic complexity is therefore dependent on the specific xLSTM configuration and implementation. A more rigorous complexity analysis would require the explicit architecture, parameter count, sequence dimensions, and computational operations associated with the implemented model.

For the present evaluation, empirical execution measurements provide a complementary indication of computational behavior. Experiment 8 achieved the highest reported classification accuracy while requiring less reported training time than Experiment 9. This combination of classification performance and measured execution behavior motivated the selection of Experiment 8 for the detailed classification analyses.

However, execution times obtained in a cloud-based environment cannot by themselves establish the suitability of the model for resource-constrained edge deployment. A deployment-oriented evaluation should additionally consider parameter count, memory requirements, inference latency, processor utilization, and energy consumption on representative edge hardware.

The experimental results provide empirical evidence that the xLSTM-based TIA achieves high DDoS classification performance on the evaluated CIC-IoT-2023 data. These findings support the effectiveness of the machine-learning component under the specified experimental conditions. They should not, however, be interpreted as end-to-end experimental validation of the complete FMAD architecture. Experimental assessment of federated coordination, inter-agent communication, distributed model aggregation, energy consumption, and complete end-to-end operation remains an important direction for future work.

## 6. Discussion

The rapid proliferation of edge–cloud IoT ecosystems has fundamentally transformed the requirements of modern cybersecurity architectures. Unlike conventional intrusion detection systems that primarily rely on centralized analysis, next-generation cyber defense platforms must support distributed intelligence, autonomous decision-making, and continuous collaboration among heterogeneous edge devices. This vision is consistent with previous studies indicating that future cyber-defense systems will increasingly depend on autonomous reasoning and automated responses operating with limited or no human intervention under rapidly evolving attack conditions [41]. Building upon this perspective, the proposed FMAD framework extends existing autonomous cyber defense paradigms by introducing intelligent agent communication as the coordination layer designed to support distributed reasoning, collaborative decision-making, and adaptive mitigation across edge–cloud IoT environments. The architectural design of FMAD and the experimental results for the TIA component suggest that intelligent agent communication should be regarded as a core architectural capability rather than merely a communication mechanism. Nevertheless, it is important to emphasize that the full multi-agent coordination workflow was not experimentally validated in this study.

Rather than proposing another standalone intrusion detection model, FMAD presents an integrated multi-agent architecture in which sensing, threat analysis, secure communication, collaborative learning, and adaptive mitigation operate as complementary functions. The Traffic Sensing Agent continuously monitors local network conditions, the Threat Intelligence Agent transforms observations into actionable security intelligence, the Adaptive Mitigation Agent coordinates response actions, the Federated Learning Agent enables privacy-preserving collaborative model optimization, and the Secure Communication Agent protects every interaction among these entities. This modular organization is intended to support scalability, interoperability, and maintainability while allowing individual agents to evolve independently without affecting the overall architecture. Consequently, FMAD provides a flexible foundation for incorporating future intelligent agents responsible for policy management, digital twins, resource orchestration, or AI-assisted decision support.

Secure communication is a fundamental requirement for collaborative autonomous cyber defense because intelligent agents continuously exchange sensitive threat intelligence, model parameters, mitigation commands, and situational awareness information. Accordingly, the proposed Secure Communication Agent combines elliptic curve cryptography with authenticated ChaCha20-Poly1305 encryption to ensure confidentiality, integrity, authentication, and replay protection while maintaining computational efficiency suitable for resource-constrained IoT devices. Compared with traditional RSA-based solutions, elliptic curve cryptography provides equivalent security using substantially smaller key sizes, thereby reducing computational overhead and communication latency. Similarly, ChaCha20-Poly1305 has demonstrated excellent software performance on low-power processors lacking dedicated AES hardware acceleration. Although emerging post-quantum cryptographic algorithms, including CRYSTALS-Kyber and CRYSTALS-Dilithium, are expected to play an increasingly important role in future intelligent edge infrastructures, the selected cryptographic mechanisms are intended to provide a balance between security and computational efficiency.

The security analysis also clarifies an important distinction between communication security and trust in participating computational entities. The proposed cryptographic mechanisms can protect messages against interception, modification, and replay, but they cannot guarantee the correctness of an update generated by an authorized agent that has been compromised. This consideration is particularly relevant to the FLA because it currently performs the aggregation of federated model updates. A compromised FLA could potentially manipulate the aggregation process or influence the global model. Therefore, the security model treats participating agents as potentially compromiseable rather than inherently trusted and identifies robust aggregation as an additional protection layer for future distributed validation.

Another important contribution of FMAD is the architectural integration of federated learning. Traditional centralized learning requires transferring large volumes of raw network traffic to cloud servers, creating privacy risks, communication overhead, and scalability limitations. The proposed Federated Learning Agent addresses these challenges by coordinating collaborative model optimization while preserving data locality. Consequently, collaborative knowledge is exchanged through model parameters rather than raw traffic data, supporting privacy preservation while enabling continuous adaptation to evolving cyber threats.

An additional architectural consideration is that the FMAD design is not intrinsically dependent on FedAvg. FedAvg is used as the baseline aggregation strategy in the current framework because it provides a simple and well-established reference point for collaborative optimization. However, the FLA aggregation layer can accommodate alternative strategies. FedProx may be considered for heterogeneous devices and non-IID data distributions, whereas coordinate-wise median, trimmed mean, or Krum may provide greater robustness when malicious or Byzantine model updates are present. This modularity allows the aggregation strategy to be selected according to the operational characteristics and threat model of the deployment environment.

The experimental evaluation demonstrated that the proposed Threat Intelligence Agent, implemented using an xLSTM-based intrusion detection model trained on the CIC-IoT-2023 dataset, achieved an overall detection accuracy of 99.89% together with very low false-positive and false-negative rates. These results indicate that xLSTM effectively captures the temporal characteristics of contemporary DDoS traffic and provides a reliable analytical engine for intelligent edge-based threat detection. Nevertheless, the principal contribution of this study is not the xLSTM classifier itself but the FMAD architecture that integrates intelligent communication, secure collaboration, and distributed intelligence into a unified autonomous cyber-defense framework.

Several limitations should nevertheless be acknowledged. First, only the Threat Intelligence Agent has been experimentally validated, whereas the remaining FMAD components are currently presented as architectural modules. Second, the secure communication, federated learning, and blockchain components have not yet been evaluated in a fully distributed edge deployment. In particular, the current study does not experimentally validate the behavior of a compromised FLA or malicious federated clients, and therefore the effectiveness of Byzantine-resilient aggregation remains an open empirical question that requires dedicated future investigation. Third, the computational and energy overhead of the complete federated workflow has not been measured on resource-constrained edge or smartphone-class devices. Although the xLSTM-based TIA demonstrates favorable training and testing times in the current experimental environment, these measurements should not be interpreted as evidence of end-to-end energy efficiency for the complete FMAD architecture. Future work should therefore evaluate the framework using heterogeneous edge devices, non-IID federated datasets, malicious-client scenarios, alternative aggregation strategies, communication latency, CPU and memory utilization, model-update size, aggregation time, and energy consumption. Such experiments would provide a more comprehensive assessment of the security, scalability, robustness, and operational feasibility of FMAD. The modular design of FMAD also provides a basis for adaptation to other distributed edge and cyber-physical environments beyond the IoT scenarios considered in this study. For example, systems involving distributed sensing, autonomous communication, and resource-constrained edge nodes could potentially employ the same agent-based architecture after adapting the sensing, communication, and mitigation modules to application-specific requirements. Such extensions, including multi-motor and unmanned aerial vehicle environments, require dedicated validation because their communication patterns, computational constraints, and threat profiles may differ from those of conventional edge–cloud IoT systems.

The architectural design and the experimental results for the TIA component suggest that intelligent agent communication should be regarded as a core architectural capability rather than merely a communication mechanism. By integrating distributed sensing, secure collaboration, privacy-preserving learning, and autonomous decision-making within a unified framework, FMAD establishes a promising architectural foundation designed to support scalable, trustworthy, and adaptive cyber defense in next-generation edge–cloud IoT ecosystems.

## 7. Conclusions

This study proposed FMAD, a conceptual modular architecture for autonomous cyber defense in edge–cloud IoT environments that integrates intelligent agent communication, distributed sensing, privacy-preserving federated learning, secure inter-agent communication, and adaptive mitigation within a unified collaborative framework. Unlike conventional intrusion detection approaches that primarily emphasize centralized analysis or standalone detection models, FMAD is designed to support distributed intelligence, collaborative decision-making, and autonomous cyber defense across heterogeneous edge–cloud IoT infrastructures.

As an initial proof of concept, the proposed TIA was implemented using an xLSTM-based intrusion detection model trained and evaluated on the CIC-IoT-2023 dataset. The experimental evaluation achieved an overall detection accuracy of 99.89%, demonstrating the capability of xLSTM to effectively capture temporal attack patterns and providing experimental evidence for the feasibility of the TIA component within the proposed FMAD architecture. The primary architectural contribution of this work is the FMAD framework, while the xLSTM-based TIA provides the empirical proof of concept evaluated in this study. By separating sensing, intelligence, communication, learning, and mitigation into specialized autonomous agents, FMAD is designed as a modular and extensible architectural foundation for next-generation distributed cybersecurity systems.

An important scope consideration is that the present empirical validation is limited to the xLSTM-based TIA component. The TSA, SCA, FLA, and AMA agents, as well as the complete federated-learning and multi-agent cooperation workflow, are evaluated at the architectural, security, and operational-design levels rather than through an end-to-end distributed deployment. Therefore, the reported experimental results demonstrate the feasibility of the proposed TIA for DDoS detection and should not be interpreted as empirical validation of the complete FMAD framework. Therefore, the reported experimental results demonstrate the effectiveness of the proposed TIA for DDoS detection and should not be interpreted as empirical validation of every FMAD component. Full-system validation involving distributed federated training, agent-to-agent communication, malicious-client scenarios, aggregation robustness, and resource-constrained edge devices remains an important direction for future work.

Future work will focus on implementing and experimentally validating the complete FMAD architecture in real distributed edge environments, with particular emphasis on intelligent agent communication, federated learning across heterogeneous edge nodes, secure agent-to-agent communication, blockchain-supported trust management, communication efficiency, computational overhead, and energy consumption.

## Figures and Tables

**Table 1:** Summary of representative DDoS detection and distributed cyber defense approaches.

Reference	Framework Type	Intelligence
Yin et al. [25]	Deep learning-based IDS	Centralized detection
Kott et al. [12]	Autonomous multi-agent defense	No federated learning or secure agent communication
McMahan et al. [16]	Federated learning	No intelligent agent coordination
Dorri et al. [32]	Blockchain-based trust	No autonomous threat detection
This Study	FMAD (conceptual)	Integrated architecture (TIA experimentally validated)

**Table 2:** Summary of selected deep learning-based DDoS detection methods from the literature.

Reference	Method	Dataset	Accuracy
Hekmati et al. [34]	Correlation-Aware Neural Networks + LSTM	Real-world IoT (4060 nodes)	81%
Jakotiya et al. [35]	Federated Learning with Neural Networks	CIC-IoT-2023	99.84%
Jony and Arnob [36]	LSTM	CIC-IoT-2023	98.75%
Ain et al. [37]	Hybrid CNN-LSTM-Autoencoder	CIC-IoT-2023	96.78%
Çekiş et al. [38]	xLSTM	Custom IP Spoofing Dataset	~99.50%
Baalia et al. [39]	xLSTM, sLSTM, mLSTM	DNP3 (SCADA)	99.50%, 99.33%, 99.42%
Naim et al. [40]	Hybrid CNN-xLSTM-XGBoost (HybxLSTM)	TON IoT	Near-perfect
The Present Study	xLSTM	CIC-IoT-2023	99.89%

**Table 3:** Security Properties, FMAD Mechanisms, and Residual Risks.

Security Property	FMAD Mechanism	Main Limitation
Confidentiality	ChaCha20-Poly1305, TLS 1.3	A compromised endpoint may expose data after decryption.
Integrity	Authenticated encryption, cryptographic verification	Cannot prevent malicious content generated by a compromised agent.
Authentication	Certificates, digital signatures	Compromised credentials may enable impersonation.
Replay Protection	Nonces, timestamps	Depends on correct nonce and timestamp management.
Traceability & Accountability	Permissioned blockchain, cryptographic identities	Records actions but does not independently verify that the recorded action was legitimate or correctly executed.
Model-Update Protection	Authenticated and encrypted transmission	Does not prevent malicious updates generated by compromised clients.
Aggregation Robustness	FedAvg; robust aggregation as an extension	Byzantine-resilient aggregation is not experimentally evaluated.

**Table 4:** Performance of the evaluated xLSTM configurations.

Experiment	Hidden Layers	Epochs	RMSE	Accuracy (%)	Loss (%)
1	2	5	0.494	50.40	49.6
2	2	15	0.464	72.57	27.43
3	2	60	0.278	87.66	12.34
4	4	5	0.454	72.80	27.2
5	4	15	0.296	91.31	8.69
6	4	60	0.145	97.71	2.29
7	6	5	0.457	75.20	24.8
8	6	15	0.108	99.89	0.4
9	6	60	0.080	99.43	0.57

## Data Availability

The CIC-IoT-2023 dataset used in this study is publicly available from the Canadian Institute for Cybersecurity (CIC): https://www.unb.ca/cic/datasets/iotdataset-2023.html (accessed on 15 June 2026).
